# Usefulness of antibody index assessment in cerebrospinal fluid from patients negative for total-IgG oligoclonal bands

**DOI:** 10.1186/2045-8118-9-14

**Published:** 2012-07-31

**Authors:** Sven Jarius, Peter Eichhorn, Brigitte Wildemann, Manfred Wick

**Affiliations:** 1 Division of Molecular Neuroimmunology, Department of Neurology, University of Heidelberg, Im Neuenheimer Feld 400, 69120 Heidelberg, Germany; 2 Department of Clinical Chemistry, Klinikum Grosshadern, Ludwig Maximilian University, Munich, Germany

**Keywords:** Intrathecal IgG synthesis, Antibody index, Cerebrospinal fluid, Oligoclonal bands, Herpes simplex virus, Cytomegalovirus, Varicella zoster virus, Measles virus, Rubella virus, Borrelia burgdorferi, Multiple sclerosis, MRZ reaction

## Abstract

**Background:**

Testing for cerebrospinal fluid (CSF)-restricted oligoclonal bands (OCB) by isoelectric focusing is used to detect intrathecally produced total IgG. By contrast, antibody indices (AI) are assessed to test for intrathecally produced antigen-specific IgG. A number of previous cases reports have suggested that AI testing might be more sensitive than OCB testing in detecting intrathecal IgG synthesis.

**Findings:**

Here we report on 21 patients with positive AI for either herpes simplex virus, varicella zoster virus, cytomegalovirus, measles virus, rubella virus, or Borrelia burgdorferi in the absence of total-IgG OCB and, accordingly, in the presence of a normal total-IgG CSF/serum ratio.

**Conclusion:**

Our findings indicate that AI testing should not generally be omitted in OCB-negative patients and provide a rationale for systematic and prospective studies on the comparative sensitivity and specificity of AI and total-IgG OCB testing in infectious and other diseases of the CNS.

## Introduction

Testing for cerebrospinal fluid (CSF)-restricted total IgG oligoclonal bands (OCB) by isoelectric focusing is a highly sensitive method for detecting intrathecal IgG synthesis, but does not take into account antibody specificity. By contrast, antibody indices (AI) are assessed to test for antigen-specific intrathecal IgG synthesis [1]. It is unclear whether AI assessment is useful in OCB negative patients. Currently, clinicians often do not test for virus- or bacterium-specific AIs on cost grounds if evidence for intrathecal total IgG synthesis as detected by OCB testing is missing.

Back in 1992, however, Felgenhauer and Reiber reported a positive varicella zoster virus (VZV)-specific AI in 6 total-IgG OCB-negative patients with VZV ganglionitis as well as in 4 total-IgG OCB-negative patients with VZV meningitis, suggesting that AI calculation might be more sensitive than total-IgG OCB testing [2]. Here, we report on 21 total-IgG OCB-negative patients with positive AI results for either herpes simplex virus (HSV), VZV, cytomegalovirus (CMV), measles virus (MV), rubella virus (RV), or Borrelia burgdorferi (Bb), thereby providing independent and corroborative evidence for Felgenhauer and Reiber’s hypothesis (see Table1 for details).

**Table 1:** Patient data and antibody indices to six viral antigens in CSF samples negative for total-IgG oligoclonal bands

**No.**	**Age**	**Sex**	**OCBs**	**AI Bb**	**AI HSV**	**AI VZV**	**AI CMV**	**AI MV**	**AI RV**	**CSF WCC**	**Suspected diagnosis and indicated by the sender**
#1A	32	M	NEG/NEG	Not det.	0.72	0.74	Not det.	0.69	0.77	1557	CSF PCR positive HSV encephalitis
#1B	32	M	NEG/NEG	Not det.	**43.40**	1.24	Not det.	ND	ND	1456	CSF PCR positive HSV encephalitis, follow-up 19 days later
#1C	32	M	NEG/NEG	ND	**53.40**	**16.30**^**#**^	ND	ND	ND	152	CSF PCR positive HSV encephalitis, follow-up a further 3 days later
#2A	33	M	NEG/NEG	Not det.	**2.02**	1.54	ND	ND	0.83	No data	Blistering rash V2, peripheral facialis paresis
#2B	33	M	NEG/NEG	ND	**1.94**	1.39	ND	ND	ND	19	Blistering rash V2, peripheral facialis paresis, follow-up 8 days later
#3	30	M	NEG/NEG	ND	**2.43**	1.12	ND	0.85	1.10	2	No data available
#4	29	F	NEG/NEG	Not det.	**2.12**	0.99	1.30	ND	0.94	3	No data available
#5A	34	F	NEG/NEG	Not det.	ND	ND	ND	ND	ND	No data	VZV meningoencephalitis
#5B	34	F	NEG/NEG	ND	Not det.	**3.99**	Not det.	ND	ND	360	VZV meningoencephalitis, follow-up 5 days later
#6	60	F	NEG/NEG	Not det.	1.20	**2.40**	0.86	ND	ND	2	“FK506-associated leukoencephalopathy”, grand mal
#7	51	M	NEG/NEG	1.26	1.11	**2.42**	1.03	0.90	1.03	1	“Fatigue and apathy”
#8	42	M	NEG/NEG	Not det.	1.31	**3.26**	ND	1.03	1.32	3	“Possible multiple sclerosis“
#9	74	F	NEG/NEG	ND	1.43	**2.30**	Not det.	ND	0.81	2	No data available
#10	50	F	NEG/NEG	ND	Not det.	**2.00**	ND	0.85	0.67	No data	No data available
#11	71	F	POS/POS*	**20.90**	1.45	1.32	Not det.	0.97	0.91	4	Neuroborreliosis, encephalitis
#12A	34	F	NEG/NEG	**2.60**	0.91	0.80	Not det.	0.70	0.80	82	Neuroborreliosis with abducens and facial nerve paresis
#12B	34	F	NEG/NEG	**1.70**	0.91	0.95	Not det.	1.15	0.83	277	Confirmatory follow-up sample obtained 5 days later
#13	44	M	NEG/NEG	**10.60**	0.90	1.00	0.88	1.5	ND	No data	"Aseptic Meningitis”
#14	84	M	NEG/NEG	**5.31**	1.02	0.99	0.85	1.02	1.04	2	No data available
#15	34	F	NEG/NEG	**4.79**	Not det.	0.99	Not det.	0.92	ND	1	No data available
#16	57	M	NEG/NEG	**3.30**	0.96	0.88	1.20	0.76	0.75	1	No data available
#17	21	M	NEG/NEG	**2.50**	Not det.	0.85	Not det.	ND	0.85	1	No data available
#18	38	F	NEG/NEG	ND	1.67	1.00	**2.40**	0.44	0.94	No data	“Encephalitis”
#19	40	F	NEG/NEG	Not det.	Not det.	**16.20**	1.43	0.72	0.90	No data	No data available
#20	25	F	NEG/NEG	Not det.	0.92	1.00	Not det.	ND	**2.10**	1	“Clinically possible multiple sclerosis (Poser)”
#21	60	M	NEG/NEG	ND	0.85	0.87	Not det.	**2.80**	0.92	No data	No data available

AI = antibody index; Bb = Borrelia burgdorferi; CMV = cytomegalovirus; HSV = herpes simplex virus; MV = measles virus; NEG/NEG = negative in CSF/negative in serum; not det. = not detectable; ND = not done; OCBs = oligoclonal bands; RV = rubella virus; VZV = varicella zoster virus. * Mirror pattern (so-called “pattern 4” according to an international consensus on OCB diagnostics) in the absence of CSF-restricted IgG bands. ^#^ Cross-reactivity between HSV and VZV as a result of epitope spreading (see reference [2]).

## Methods and results

Testing for OCB was performed by a CSF laboratory with long-standing expertise in the field that takes part in the official German external quality assessment organized by INSTAND e.V. twice a year. OCB were determined by isoelectric focusing followed by anti-IgG immunofixation (Sebia, Fulda, Germany). Serum and CSF levels of antibodies to HSV, VZV, CMV, MV, RV, and Bb were assessed using commercially available enzyme linked immunosorbent assays (Enzygnost, Siemes Healthcare, Eschborn, Germany). Total-IgG and total albumin concentrations in CSF and serum were determined nephelometrically (BN ProSpec, Dade Behring, Germany). Intrathecal synthesis of IgG to HSV, VZV, CMV, MV, RV, and Bb was determined by calculating the respective antibody indices (AI): AI=QIgG[spec]/QIgG[total], if QIgG[total]<Qlim, and AI=QIgG[spec]/Qlim, if QIgG[total]>Qlim, with QIgG[spec]=IgGspec[CSF]/IgGspec[serum], and QIgG[total]=IgGtotal[CSF]/IgGtotal[serum][1,3]. The upper reference range of QIgG[total], Qlim, was calculated according to Reiber’s formula [1]. No lumbar puncture or phlebotomy was performed for this study, and no stored CSF or serum samples were used for this study. Instead, all data were retrieved retrospectively by an automated database search and analyzed anonymously. As clinical data were not available from all patients due to anonymization, a very conservative cut-off for AI positivity (2.0 instead of 1.3) was applied to preclude false-positive results. No other inclusion and exclusion criteria than OCB negativity and presence of a positive AI for any of those six viral and bacterial antigens in the same paired CSF/serum sample were applied. A positive AI was present for Bb in 7 cases (2.5-20.9; median, 4.794) for HSV in 4 cases (2.02-43.4; 2.28), for VZV in 7 (2–16.2; 2.42), for CMV in 1 (2.40), for MV in 1 (2.80), and for RV in 1 (2.10)**.** In case #1, HSV encephalitis was confirmed by PCR and a follow-up lumbar puncture (LP) confirmed the positive AI, again in the absence of total-IgG OCB. In a second case with positive HSV-AI and negative total-IgG OCB, herpes simplex blisters in the area innervated by the second branch of the trigeminal nerve were present and the patient developed facial nerve palsy; the result was confirmed in a second CSF/serum samples obtained 8 days later. Confirmation from repeat lumbar puncture was also available in a case of neuroborreliosis (Table1). All of these cases were associated with CSF pleocytosis. Other clinical diagnoses associated with positive viral AIs as provided by the senders included “encephalitis”, “meningitis”, “abducens and facial nerve paresis“, and “clinically possible multiple sclerosis”; one VZV-AI positive patient was treated with tacrolimus (FK506), a potent immunosuppressant, at the time of LP (Table1).

**Figure 1:**
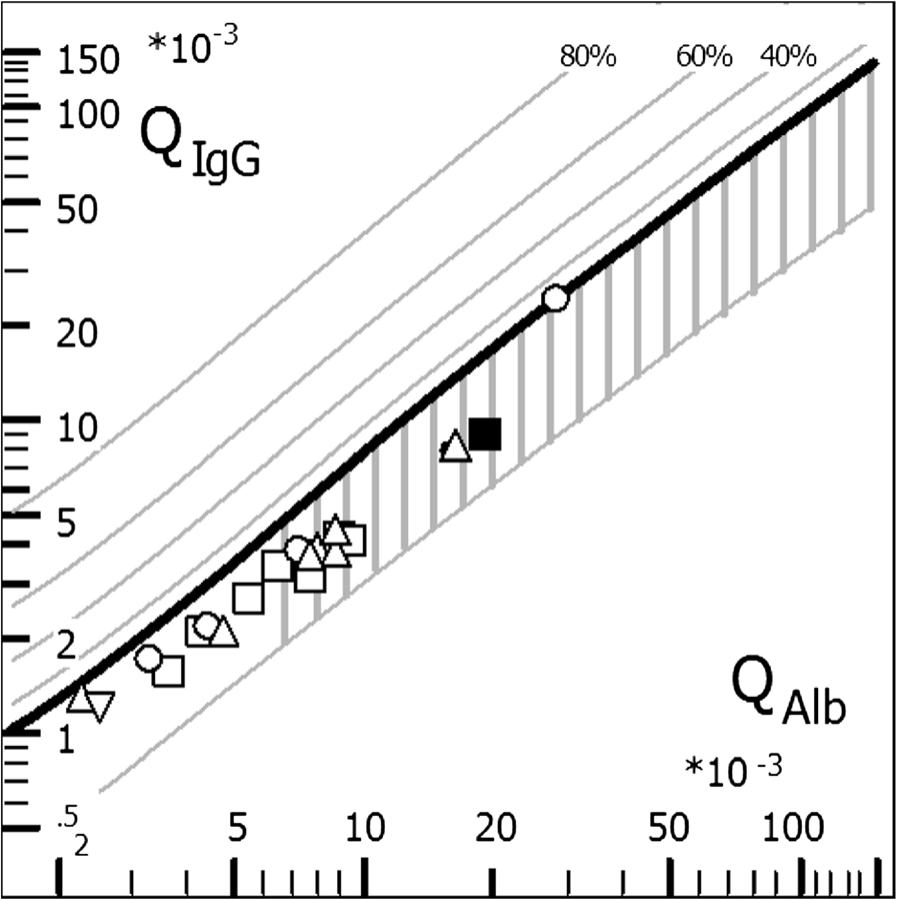
** CSF/serum quotient diagram for IgG (‘Reibergram’).** Individual CSF/serum ratios of IgG (QIgG) were plotted against CSF/serum albumin ratios (QAlb). Values above the upper hyperbolic discrimination line Qlim indicate intrathecal total-IgG synthesis. Individual intrathecal fractions, IgIF, can be obtained by interpolation from the percentiles above Qlim. The Reibergram plot indicated that there was no intrathecal total-IgG synthesis in the patients tested here, confirming the negative OCB results. IgG = immunoglobulin G; QIgG = CSF/serum total-IgG ratio; QAlb = CSF/serum albumin ratio. Graph created using CSF Research Tool, Comed, Germany**.** Open squares: Borrelia burgdorferi, Bb; upward triangles: varicella zoster virus, VZV; open circles: herpes simplex virus, HSV; filled circles: cytomegalovirus, CMV; filled squares: measles virus, MV; downward triangles: rubella virus, RV.

To assess the plausibility of the negative total-IgG OCB results, we analysed each patients’ total-IgG CSF/serum ratio (QIgG). Elevated QIgG in the absence of total-IgG OCB positivity would suggest a false-negative OCB result. However, QIgG was found to be normal in all patients (i.e. QIgG<Qlim) as shown in Figure1.

## Discussion

These cases confirm that negative total-IgG OCBs in CSF samples might not always predict the absence of positive virus- or bacterium-specific IgG-AIs in patients with suspected CNS infection. Omitting AI testing in OCB negative patients might thus be a possible diagnostic pitfall. In addition to VZV infection [2], positive AIs have previously also been reported in individual total-IgG OCB-negative patients with autoimmune disorders of the central nervous system including anti-GAD antibody positive stiff person syndrome [4], anti-Ri positive paraneoplastic neurological syndromes (PNS) [5], anti-CV2/CRMP5 positive PNS [6], and anti-Yo positive PNS [3].

Around 2-5 % of patients with MS are negative for OCBs. This subgroup, which is small in relative numbers but not so small in absolute numbers due to the high prevalence of MS, is particularly challenging from a diagnostic point of view. A higher sensitivity of antigen-specific AI calculation compared to total-IgG OCB testing might therefore also be of potential relevance for the laboratory diagnosis of multiple sclerosis (MS), since a majority of patients with MS display a polyspecific humoral antibody response to a broad variety of viral and bacterial antigens as determined by AI calculation, with intrathecal antibodies to MV, RV and VZV as its most common constituents (termed MRZ reaction or MRZR), as demonstrated by Reiber and others [7-9]. Apart from MS, a positive MRZR reaction has also been reported in rare cases of lupus and CNS involvement [7]. MRZR testing is currently performed by many CSF laboratories in Germany, but, in our experience, is so far almost exclusively done in OCB-positive patients. In line with the hypothesis that tests for antigen-specific IgG synthesis might be more sensitive than total-IgG OCB detection, Stich et al. recently reported on positive AIs for MV, RV, and VZV in 2/17 (12 %) total-IgG OCB-negative patients with MS but in none of 11 controls. Similarly, the authors found MV-, RV-, and VZV-specific bands in 3/17 (18 %) total-IgG OCB-negative patients with MS as detected by affinity blotting using recombinant viral antigens and a highly sensitive chemiluminescence detection technique [8]. This is in accordance with a report by Frederiksen and Sindic (1998), who found CSF-restricted VZV- and mumps-specific bands in total-IgG OCB-negative patients with MS using a similar type of assay [9].

On the other hand, the fact that a subset of patients with MS presents with a monospecific AI elevation rather than the typical polyspecific, oligoclonal reaction, at least at first presentation [10], and that the spectrum of positive AIs can be broader than just MV, RV, and VZV and include (though less frequently) positive AIs for Bb, toxoplasma and other viral and bacterial agents can pose differential diagnostic challenges in some cases. Accordingly, in patients in whom MS is a reasonable differential diagnosis, AIs for at least the three main constituents of the MRZ reaction (MV, RV, and VZV) should be determined (approximate costs in Germany, ~20-30 €/AI determination). Moreover, AI results – as laboratory results in general – must always be interpreted in the context of the patient’s overall clinical and neuroradiological presentation and the patient’s CSF findings such as white cell count and differentiation, CSF/serum total-IgM ratios or total-IgA ratios before a definite diagnosis can be made [1,11]. Bb-IgM assessment can be useful in patients with suspected acute Bb infection; however, it was negative in 6/6 patients in our study (not determined in one).

The reason for the higher sensitivity of AI detection compared to total-IgG detection in the patients reported here is unknown. Infections are often associated with severe blood-CSF barrier dysfunction; the presence of OCB may become undetectable in those cases because of a relative increase of the blood-derived polyclonal IgG fraction. In such cases OCBs may possibly become positive after normalization of barrier dysfunction. However, high CSF/serum albumin ratios were present only in a subset of our patients (see Figure1). Moreover, the higher sensitivity of antigen-specific OCBs compared to total-IgG OCBs detection as demonstrated in a number of studies [3-5] argues in favour of additional, so far unidentified factors specific for total-IgG OCB detection rather than OCB detection in general.

Possibly, AIs might become positive earlier than total-IgG OCBs. If so, AI assessment might be particularly useful at disease onset. However, as OCBs remained negative in the few patients with follow-up LPs in our study, our study provides no direct evidence for this hypothesis.

There are some caveats. First, as it is the case with IgG responses in general, there might be a delay in AIs becoming positive as indicated by case 1 in Table1; this patients became positive for HSV only at lumbar punctures (LP) #2 and LP #3. However, similar problems apply to HSV PCR, which is sometimes negative at first LP and becomes positive only with time. Therefore, repeat LP is recommended if the initial LP is negative. Second, an expansion of the polyspecific, intrathecal immune response in MS over time has been described [10]. Accordingly, repeat LP might possibly be useful also in individual patients with suspected MS but missing MRZR reaction at first LP. Third, cross-reactivity between VZV and HSV is a well-known phenomenon. However, the true-positive AI generally yields higher values, which usually allows identifying the relevant agent (see patient 1, LP #3). It is therefore recommended to test always for both VZV-AI and HSV-AI. Third, the 21 cases presented here were collected over a period of 9 years, suggesting that the phenomenon is relatively rare; however, it is potentially of high relevance for the individual patient.

In summary, the cases reported here indicate that AI testing should not generally be omitted in total-IgG OCB-negative patients and provides a rationale for systematic and prospective studies on the comparative sensitivity and specificity of AI and total-IgG OCB testing in infectious diseases of the CNS as well as in other indications.

## Abbreviations

AI: antibody index; Bb: Borrelia burgdorferi; CSF, cerebrospinal fluid; CMV: cytomegalovirus; CNS: central nervous system; HSV: herpes simplex virus; IgG: immunoglobulin G; LP: lumbar puncture; MRZR: measles virus, rubella virus, and varicella zoster virus reaction; MS: multiple sclerosis; MV: measles virus; NB: neuroborreliosis; OCB: oligoclonal IgG bands; PCR: polymerase chain reaction; PNS: paraneoplastic neurological syndromes; Q: ratio; RV: rubella virus; VZV: varicella zoster virus; WCC: white cell count.

## Competing interests

The authors declare that they have no competing interests.

## Authors’ contributions

S.J. conceived the study, analysed the data, and wrote the initial draft; P.E. and M.W. conducted and interpreted the laboratory tests; S.J., P.E., B.W., and M.E. revised the manuscript for important intellectual content. All authors have read and approved the final version of the manuscript.
